# Evaluation of the DOAC-Stop® Procedure to Overcome the Effect of DOACs on Several Thrombophilia Screening Tests

**DOI:** 10.1055/s-0038-1657785

**Published:** 2018-06-01

**Authors:** Julien Favresse, Benjamin Lardinois, Lina Sabor, Bérangère Devalet, Julie Vandepapeliere, Maximilien Braibant, Sarah Lessire, Bernard Chatelain, Hugues Jacqmin, Jonathan Douxfils, François Mullier

**Affiliations:** 1 Université Catholique de Louvain, CHU UCL Namur, Hematology Laboratory, Namur Thrombosis and Hemostasis Center, NARILIS, Yvoir, Belgium; 2 Department of Hematology, CHU UCL Namur, Namur Thrombosis and Hemostasis Center, Université Catholique de Louvain, Yvoir, Belgium; 3 Department of Pharmacy, CHU UCL Namur, Namur Thrombosis and Hemostasis Center, Université Catholique de Louvain, Yvoir, Belgium; 4 Department of Anesthesiology, CHU UCL Namur, Namur Thrombosis and Hemostasis Center, Université Catholique de Louvain, Yvoir, Belgium; 5 Department of Pharmacy, Namur Thrombosis and Hemostasis Center, Université de Namur, Yvoir, Belgium; 6 QUALIblood SA, Namur, Belgium

**Keywords:** thrombophilia, direct oral anticoagulants, interference

## Abstract

The impact of direct oral anticoagulants (DOACs) on laboratory assays used for thrombophilia testing (e.g., antithrombin, protein S, protein C, lupus anticoagulant and activated protein-C resistance) is a well-known issue and may cause false-positive and -negative results. Therefore, the correct interpretation of tests that are performed in patients taking DOACs is mandatory to prevent misclassification and the subsequent clinical consequences. We aimed at evaluating the efficiency of a new and simple procedure (DOAC-Stop®; Haematex Research, Hornsby, Australia) to overcome the effect of all DOACs in real-life settings and to assess the percentage of erroneous results due to the presence of DOACs on thrombophilia screening tests. For this purpose, 135 DOAC-treated patients (38 apixaban, 40 dabigatran, 15 edoxaban, and 42 rivaroxaban) and 20 control patients were enrolled. A significant drop in apixaban, dabigatran, edoxaban, and rivaroxaban plasma concentrations following the DOAC-Stop® treatment was observed (74.8–8.2 ng/mL [ *p* < 0.0001], 95.9–4.7 ng/mL [ *p* < 0.0001], 102.1–8.8 ng/mL [ *p* = 0.001], and 111.3–7.0 ng/mL [ *p* < 0.0001], respectively). The DOAC-Stop® treatment was mostly effective to overcome the effect of DOACs on PTT-LA, dilute Russell's viper venom time (dRVVT) screen, and dRVVT confirm tests. Using our procedures, false-positive results due to DOACs were observed only with lupus anticoagulant tests (up to 75%) and fell to zero after the DOAC-Stop® procedure, regardless of the DOAC considered. In conclusion, the DOAC-Stop® adsorbent procedure appeared to be an effective and simple way to overcome the interference of DOAC on coagulation tests and should facilitate the interpretation of thrombophilia screening tests in patients taking DOACs.

## Introduction

Direct oral anticoagulants (DOACs) including apixaban, dabigatran etexilate, edoxaban, and rivaroxaban are used worldwide since their approval in several thromboembolic disorders, including the treatment and secondary prevention of recurrent venous thromboembolism (VTE) and pulmonary embolism (PE) as well as the prevention of stroke and systemic embolism in patients with nonvalvular atrial fibrillation (NVAF). 1 2 3 4

The impact of DOACs on laboratory assays used for thrombophilia testing (e.g., antithrombin, protein S, protein C, lupus anticoagulant, and activated protein-C resistance [APC-R]) is a well-known issue and may cause false-positive and -negative results. 5 6 7 8 9 Therefore, the correct interpretation of results that are performed in patient taking DOACs is mandatory to prevent misclassification and the subsequent clinical consequences. 7

Several strategies were proposed to minimize the impact of residual DOACs on coagulation assays: (1) the use of DOAC-insensitive assays, (2) the addition of idarucizumab to the plasma sample (Praxbind, Boehringer Ingelheim) to specifically neutralize the in vitro activity of dabigatran, 10 or (3) missing one (for once-daily fixed-dose regimens) or two (for twice-daily fixed-dose regimens) DOAC intake in patients with low thromboembolic risk. 6 However, any interruption of anticoagulation will expose the patient to an increased risk of thrombosis and residual drug levels may still affect test results. 7 Thus, none of these approaches are considered optimal and a simple way to overcome the problem would be to remove DOAC from the plasma sample without influencing its coagulant property.

The aim of this study was to evaluate the efficiency of a new and simple procedure (DOAC-Stop®; Haematex Research, Hornsby, Australia) 11 to overcome the effect of all DOACs in real-life settings and to assess the percentage of erroneous results due to the presence of DOACs on thrombophilia screening tests.

## Materials and Methods

### Plasma Samples

The study protocol was in accordance with the Declaration of Helsinki and was approved by the Medical Ethical Committee of the CHU UCL Namur, Université Catholique de Louvain (Yvoir, Belgium, approval number 31/2016). Plasma samples from 135 DOAC-treated patients (38 apixaban, 40 dabigatran, 15 edoxaban, and 42 rivaroxaban) and from 20 patients without any anticoagulant (controls) were collected between August 2014 and January 2018. The study population displayed the following characteristics: 73 females and 82 males aged 20 to 92 years (mean age = 70 years). None of these patients were known to be LA positive. Blood was taken by venipuncture in the antecubital vein and collected into 0.109 M sodium citrate (9:1 v/v) tubes (Vacuette, Greiner Bio-One, Courtaboeuf, France) using a 21-gauge needle (Greiner Bio-One). Platelet-poor plasma (PPP) was obtained from the supernatant fraction of the blood tubes after a double centrifugation for 15 minutes at 2,000 *g* at room temperature. Immediately after centrifugation, PPP from the 155 patients were frozen at –80°C. Samples were thawed and heated to 37°C for 5 minutes just before experiment.

### Thrombophilia Testing

Protein S (antigenic assay, STA-Liatest Free Protein S; Diagnostica Stago, Asnières, France), protein C (chromogenic assay, STA-Stachrom Protein C, Diagnostica Stago), antithrombin activity (thrombin based-assay, STA-Stachrom AT III, Diagnostica Stago), and APC-R (Pefakit APC-R factor V Leiden using factor V–deficient plasma; DSM, Basel, Switzerland) were assayed on a STA-R MAX analyzer (Diagnostica Stago). The dilute Russell's viper venom time (dRVVT) screen and confirm (STA-Staclot dRVV Screen and Confirm, Diagnostica Stago) and the aPTT sensitive to lupus anticoagulants (PTT-LA, Diagnostica Stago) were assayed on the KC10 coagulometer (Amelung GmbH, Lemgo, Germany). LA was defined as (1) a prolongation of screening tests (low phospholipid [PL] concentration; dRVVT and/or PTT-LA), (2) a partial or no correction of the prolonged clotting time after mixing patient's plasma with normal pool plasma, and (3) a decrease in the prolonged clotting time with a confirmatory test (high PL concentration). 12 13 A dRVVT screen/dRVVT confirm ratio was also calculated. The following cutoff values were used for LA screening: PTT-LA = 41 seconds (s); dRVVT screen = 44 seconds; and [dRVVT screen/dRVVT confirm ratio] = 1.2. Normal ranges for protein S, protein C, antithrombin activity, and APC-R were 50 to 134% for women and 70 to 148% for men, 70 to 120%, 80 to 120%, and >2, respectively. 14 15

### Direct Oral Anticoagulant Measurement

Concentrations of dabigatran were estimated using one Ecarin chromogenic assay (STA-ECA-II, Diagnostica Stago), and concentrations of apixaban, edoxaban, and rivaroxaban with the corresponding procedure using the chromogenic assay (STA-liquid anti-Xa, Diagnostica Stago). All these procedures were performed on the STA-R MAX analyzer (Diagnostica Stago). The limit of quantification of the corresponding apixaban, dabigatran, edoxaban, and rivaroxaban assays were 15, 27, 20, and 25 ng/mL, respectively. 16 17 18 19

### DOAC-Stop® Treatment

Thrombophilia screening tests were performed before and after the addition of adsorbent tablets (DOAC-Stop®; Haematex Research, Hornsby, Australia), according to the manufacturer's instructions and depending on the available plasma volume. 11 Briefly, one tablet of DOAC-Stop® designed to adsorb DOACs is added to 1 mL of plasma. Thereafter, the sample is gently mixed for 5 minutes and spun for 2 minutes at 2,000 *g* to precipitate DOACs with these adsorbent tablets. Finally, the supernatant supposed to be free from DOACs is collected to be further analyzed. The composition of DOAC-Stop® is Haematex proprietary information. Concentrations of apixaban, dabigatran, edoxaban, and rivaroxaban were also assayed before and after the DOAC-Stop® procedure. The DOAC-Stop® treatment in plasma samples without DOAC or anti-Xa activity was found to minimally affect the aPTT and PT. 11 20

### Statistical Analysis

False-positive LA results caused by DOACs were compared with the results obtained after the DOAC-Stop® treatment. A paired *t* -test was used in case of passed normality test and a Wilcoxon matched-pairs signed rank test in case of failed normality test (D'Agostino–Pearson normality test). A *p* -value <0.05 was considered as statistically significant. The concept of reference change value (RCV) used to detect minimal difference between two measurements was also employed to assess significant clinical changes after the DOAC-Stop® treatment. The RCV combined the analytical imprecision (CV _A_ ) and the intraindividual coefficient of variation (CV _I_ ) and was calculated by the following equation: [RCV = 2 ^1/2^ × Z (1.96 for a probability of 95%) × (CV _A_^2^ + CV _I_^2^ ) ^1/2^ ]. 21 Biological variation data (CV _I_ ) were found in the literature. 22 23 GraphPad Prism 6.0e (California, United States) was used to perform statistical analysis.

## Results

**Fig. 1:**
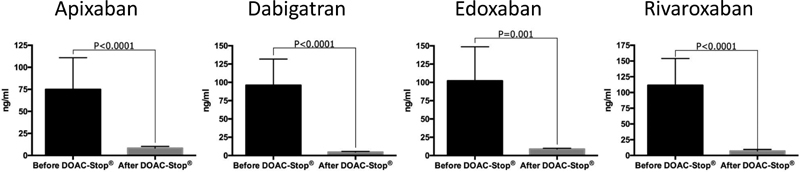
Impact of the DOAC-Stop® adsorbent treatment on apixaban, dabigatran, edoxaban, and rivaroxaban concentrations. The mean (and 95% confidence interval) of each direct oral anticoagulant is presented before and after the DOAC-Stop® treatment.

Plasma concentrations of apixaban, dabigatran, edoxaban, and rivaroxaban before DOAC-Stop® treatment varied from 10 to 316 ng/mL, 2 to 406 ng/mL, 21 to 354 ng/mL, and 7 to 456 ng/mL, respectively. A significant drop in apixaban, dabigatran, edoxaban, and rivaroxaban concentrations following the DOAC-Stop® treatment was also observed (from 74.8 to 8.2 ng/mL [ *p* < 0.0001], from 95.9 to 4.7 ng/mL [ *p* < 0.0001], from 102.1 to 8.8 ng/mL [ *p* = 0.001], and from 111.3 to 7.0 ng/mL [ *p* < 0.0001], respectively; Fig. 1 ). The residual level of each DOAC was lower than the limit of quantification of the corresponding apixaban, dabigatran, edoxaban, and rivaroxaban assays. 16 17 18 19

**Fig. 2:**
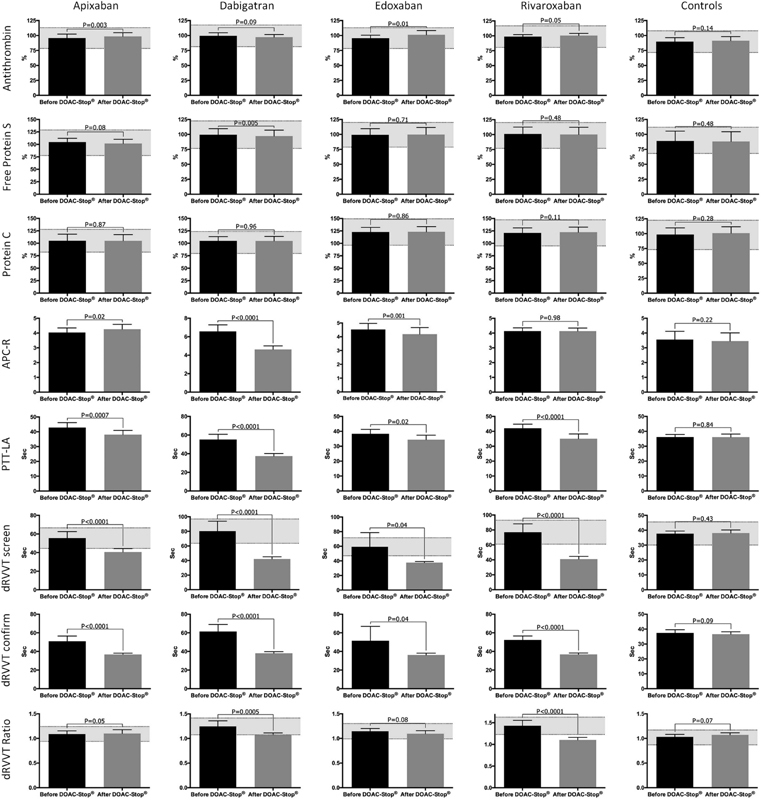
Impact of the DOAC-Stop® adsorbent treatment on common thrombophilia screening tests. The gray zone corresponds to the reference change value interval of each test. The mean (and 95% confidence interval) of each parameter is presented before and after the DOAC-Stop® treatment.

The DOAC-Stop® treatment was mostly effective to overcome the effect of DOACs on PTT-LA, dRVVT screen, and dRVVT confirm tests ( Table 1 and Fig. 2 ). False-positive results were observed only with LA tests (up to 75%) and fell to zero after the DOAC-Stop® procedure, regardless of the DOAC considered ( Table 2 ).

**Table 1:** Impact of the DOAC-Stop® adsorbent treatment on common thrombophilia screening tests

	Apixaban		Dabigatran		Edoxaban		Rivaroxaban		Controls	
	80 ng/mL (10–316)		73.5 ng/mL (2–406)		136.5 ng/mL (21–354)		76.5 ng/mL (7–456)			
	Before DOAC-Stop®	After DOAC-Stop®	Before DOAC-Stop®	After DOAC-Stop®	Before DOAC-Stop®	After DOAC-Stop®	Before DOAC-Stop®	After DOAC-Stop®	Before DOAC-Stop®	After DOAC-Stop®
Antithrombin (%)	95.7	98.6	99.5	97.2	95.6	101.3	98.7	100.3	89.8	91.4
	*p* = 0.003 ( *n* = 26) a		*p* = 0.09 ( *n* = 30)		*p* = 0.01 ( *n* = 10) a		*p* = 0.05 ( *n* = 27)		*p* = 0.14 ( *n* = 19)	
Free protein S (%)	104.5	101.7	99.58	97.26	99.3	99.8	101	100.2	89.2	88.3
	*p* = 0.08 ( *n* = 24)		*p* = 0.005 ( *n* = 31) a		*p* = 0.71 ( *n* = 10)		*p* = 0.48 ( *n* = 25)		*p* = 0.48 ( *n* = 19)	
Protein C (%)	105	104.9	104.8	104.7	122.7	123.2	121.0	122.5	98.5	100.8
	*p* = 0.87 ( *n* = 24)		*p* = 0.96 ( *n* = 32)		*p* = 0.86 ( *n* = 10)		*p* = 0.11 ( *n* = 25)		*p* = 0.28 ( *n* = 19)	
APC-R	4.0	4.3	6.6	4.6	4.5	4.2	4.1	4.1	3.6	3.5
	*p* = 0.02 ( *n* = 20) a		*p* < 0.0001 ( *n* = 30) a		*p* = 0.001 ( *n* = 10) a		*p* = 0.98 ( *n* = 26)		*p* = 0.22 ( *n* = 19)	
PTT-LA (s)	42.8	38.1	55.3	37.5	38.3	34.4	42.1	35.1	36.2	36.1
	*p* = 0.0007 ( *n* = 26) a		*p* < 0.0001 ( *n* = 27) a		*p* = 0.02 ( *n* = 8) a		*p* < 0.0001 ( *n* = 31) a		*p* = 0.84 ( *n* = 19)	
dRVVT screen (s)	55.5	40.6	80.2	42.1	59.3	37.7	77.0	40.9	37.7	38.1
	*p* < 0.0001 ( *n* = 27) a		*p* < 0.0001 ( *n* = 31) a		*p* = 0.04 ( *n* = 8) a		*p* < 0.0001 ( *n* = 31) a		*p* = 0.43 ( *n* = 18)	
dRVVT confirm (s)	49.90	36.8	61.4	38.0	51.5	36.2	52.3	36.8	37.4	36.6
	*p* < 0.0001 ( *n* = 27) a		*p* < 0.0001 ( *n* = 29) a		*p* = 0.04 ( *n* = 8) a		*p* < 0.0001 ( *n* = 31) a		*p* = 0.09 ( *n* = 20)	
dRVVT ratio	1.1	1.1	1.2	1.1	1.1	1.1	1.4	1.1	1.0	1.1
	*p* = 0.05 ( *n* = 27)		*p* = 0.0005 ( *n* = 27) a		*p* = 0.08 ( *n* = 8)		*p* < 0.0001 ( *n* = 31) a		*p* = 0.07 ( *n* = 18)	

Abbreviations: APC-R, activated protein-C resistance; dRVVT, dilute Russell's viper venom time.

Notes: The minimal, median, and maximal DOAC concentration is indicated in the first line of the table. The mean (and 95% confidence interval) of each parameter is presented before and after the DOAC-Stop® treatment.

a *p* -Value <0.05.

**Table 2:** False-positive LA results caused by DOACs

	Apixaban	Dabigatran	Edoxaban	Rivaroxaban	Controls
	80 ng/mL (10–316)	73.5 ng/mL (2–406)	136.5 ng/mL (21–354)	76.5 ng/mL (7–456)	
PTT-LA (s)	8/26 (30.8%) From 26 ng/mL	19/27 (70.4%) From 33 ng/mL	2/8 (25%) From 43 ng/mL	11/31 (35.5%) From 23 ng/mL	0/19 (0%)
dRVVT screen (s)	11/27 (40.7%) From 20 ng/mL	22/31 (71.0%) From 23 ng/mL	6/8 (75%) From 21 ng/mL	20/31 (64.5%) From 54 ng/mL	0/18 (0%)
dRVVT ratio	1/27 (3.7%) From 20 ng/mL	7/27 (26%) From 16 ng/mL	1/8 (12.5%) From 199 ng/mL	16/31 (51.6%) From 65 ng/mL	0/18 (0%)

Abbreviation: dRVVT, dilute Russell's viper venom time.

Notes: The following cutoffs were used to calculate the number of false-positives values: PTT-LA = 41 seconds; dRVVT screen = 44 seconds; and [dRVVT screen/dRVVT confirm ratio] = 1.2.

Significant differences following the DOAC-Stop® procedure were also observed for the protein S (dabigatran, *p* = 0.005) and antithrombin activity (apixaban and edoxaban, *p* = 0.03 and *p* = 0.01). However, differences obtained before and after the DOAC-Stop® treatment were always below the RCV, whatever the DOAC tested. The clinical impact is therefore not relevant ( Fig. 2 ).

Except for rivaroxaban ( *p* = 0.98), a significant difference was observed for APC-R. The most obvious difference was pointed out in the dabigatran group (mean absolute difference of 2.0).

No significant changes were observed in the control group.

## Discussion

Our observations confirmed that APC-R, PTT-LA, dRVVT screen, dRVVT confirm, and dRVVT ratio could be influenced by DOACs and that false-positive LA results were encountered with all DOACs. 5 6 7 24 The DOAC-Stop® treatment appeared to be an effective way to overcome the effect of DOACs on these aforementioned tests. Furthermore, the DOAC-Stop® procedure did not significantly affect all thrombophilia screening tests in control patients.

The impact of DOACs on these tests is a well-known issue, but the concentrations at which thrombophilia assays are impacted are not well defined yet and few data are available for edoxaban. Nevertheless, it is not recommended to perform LA testing in patients taking DOACs, as these may cause false-positive results in a concentration-dependent manner. 8 25 It is recommended to stop taking DOACs at the time of thrombophilia testing (for at least 2 days or at trough concentration) to overcome the interference. 6 7 25 26 However, the discontinuation of the treatment is not clinically recommended 7 and a high variability of DOACs trough levels has been reported. 27 Moreover, patients may also choose not to stop their DOAC treatment (or forget to stop) before thrombophilia testing, which could lead to wrong diagnosis and clinical consequences. 7 9 Some authors recommended to detect LA using taipan snake venom time (TSVT), Ecarin clotting time (ECT), or textarin clotting time (TCT) as they are not affected by anti-Xa drugs. 7 25 28 29 30 However, these alternatives are not readily available in clinical practice 6 7 and are only appropriate for anti-Xa drugs, thus requiring different procedures depending on the drug.

Our results showed that DOACs had a higher impact on the dRVVT screen assay compared with PTT-LA and dRVVT ratio. First, false-positive results were already encountered at concentration as low as 20, 23, 21, and 54 ng/mL of apixaban, dabigatran, edoxaban, and rivaroxaban, respectively. Second, the mean relative decrease following the DOAC-Stop® treatment was higher for dRVVT screen (26.8, 47.5, 36.4, and 46.9% for apixaban, dabigatran, edoxaban, and rivaroxaban, respectively) compared with PTT-LA (11.0, 32.2, 10.2, and 16.6%) and dRVVT confirm (26.3, 38.1, 29.7, and 29.6%; Tables 1 and 2 ).

Third, the number of false-positive values was higher for dRVVT screen compared with PTT-LA and dRVVT ratio whatever the DOAC considered ( Table 2 ). These observations are consistent with recent publications showing that the dRVVT screen is the most sensitive test to the presence of DOACs. 24 25 False-positive results for all LA tests (PTT-LA, dRVVT screen, dRVVT confirm, and dRVVT ratio) were encountered regardless of the DOAC considered. Except for rivaroxaban, differences between dRVVT ratio values before and after the DOAC-Stop® treatment (dRVVT screen/dRVVT confirm) were all included in the RCV interval found in the literature. This must be explained by the fact that both dRVVT screen and dRVVT confirm are influenced by DOAC's presence in a proportional way. Therefore, a normal dRVVT ratio does not exclude the interference of DOAC.

Proteins S and C may be measured by three different methods: clot-based, antigenic, or chromogenic. Only clot-based methods would be affected by DOACs. 5 6 8 24 As we have used an antigenic assay for protein S and a chromogenic assay for protein C, it explains that the results were not overestimated before the DOAC-Stop® treatment. It is also reassuring to note the absence of significant difference after the DOAC-Stop® procedure when applied on the controls which suggests that this product could be used without affecting both proteins S and C. This information is useful for practical reasons, as we showed that the DOAC-Stop® treatment does not require slitting the sample into two aliquots (one treated and the other not treated by DOAC-Stop®).

The APC-R methods based on the aPTT and using factor V–deficient plasma are mostly affected by all DOACs. 5 6 24 31 However, it is known that rivaroxaban does not interfere with the Pefakit APC-R factor V Leiden (prothrombinase-based assay) used in this study, 6 32 33 while the dabigatran and edoxaban do. 8 34 35 Accordingly, we found a significant decrease following the DOAC-Stop® procedure for dabigatran (ratio from 6.6 to 4.6, *p* < 0.0001) and to a lesser extent for edoxaban (ratio from 4.5 to 4.2, *p* = 0.001). No significant decrease was found for rivaroxaban ( Table 1 and Fig. 2 ). Interestingly, the DOAC-Stop® treatment did not decrease the APC-R of patients taking apixaban.

The antithrombin activity is not affected by the presence of direct Xa inhibitors (apixaban, edoxaban, and rivaroxaban) when measured with a thrombin-based assay. 5 6 7 36 As observed for protein S and C, the DOAC-Stop® procedure did not significantly affect the antithrombin activity given that we used a thrombin-based assay. 5 6 7 36 Although we used a thrombin-based antithrombin assay, no significant difference for dabigatran following the DOAC-Stop® treatment was observed. According to the in vitro study of Douxfils et al, dabigatran started to interfere with antithrombin at higher concentration (>100 ng/mL) in a thrombin-based assay. 37 The median concentration of our patient samples for dabigatran was quite low (73.5 ng/mL) and may therefore explain the absence of significant difference after the DOAC-Stop® procedure.

Thus, compared with the use of idarucizumab (Praxbind, Boehringer Ingelheim) to overcome the in vitro effect of dabigatran or the use of other alternatives for LA screening tests (e.g., TSVT, ECT, or TCT), the DOAC-Stop® procedure has the advantage to remove all types of DOACs, to be simple, cheap, and easily accessible.

Our study has some limitations. Our population did not contain factor V Leiden carriers, LA-positive patients, and deficient in protein S and C. It would be useful to determine if the DOAC-Stop® treatment may have an impact on these particular patients. However, vitamin K antagonists are the treatment of choice for antiphospholipid syndrome, protein S or protein C deficiency, while DOAC's use is still controversial. 38 39 40 41 Additional studies designed to evaluate the efficiency of DOAC-Stop® treatment with a larger range of DOAC's concentration on more coagulation tests (including clotting factors and instruments using optical clot detection) will also be useful. Biological variation data (CV _I_ ) obtained from the publication of de Maat et al were determined with the same reagents and on a similar platform (STA Compact analyzer, Diagnostica Stago). 23 However, the CV _I_ provided by Shou et al were obtained with different settings (ACLTOP 700 analyzer, Instrumentation Laboratory, Bedford, MA). 22 Therefore, the RCV used in this study for LA tests may vary for other platforms. Nevertheless, we did not show any false-positive PTT-LA, dRVVT screen, and dRVVT confirm results following the DOAC-Stop® procedure. Information on biological variation in hemostasis variables (e.g., APC-R, PTT-LA, and dRVVT confirm) is still limited and need further evaluations, 23 but these results are clearly encouraging.

## Conclusion

This real-life study confirms that thrombophilia assays are frequently influenced by the presence of DOACs. The importance depends on the DOAC and on the assay considered. Moreover, the DOAC-Stop® adsorbent procedure appeared to be an effective and simple way to overcome the interference of DOAC on coagulation tests. Our findings should facilitate the interpretation of thrombophilia screening tests in patients taking DOACs and we suggest the use of the DOAC-Stop® treatment in clinical practice to avoid potential misclassifications and clinical consequences.
